# Electrocatalysis of Methanol Oxidation in Alkaline Electrolytes over Novel Amorphous Fe/Ni Biphosphate Material Prepared by Different Techniques

**DOI:** 10.3390/nano12193429

**Published:** 2022-09-30

**Authors:** Mai M. Khalaf, Hany M. Abd El-Lateef, Van-Duong Dao, Ibrahim M. A. Mohamed

**Affiliations:** 1 Department of Chemistry, College of Science, King Faisal University, Al-Ahsa 31982, Saudi Arabia; 2 Department of Chemistry, Faculty of Science, Sohag University, Sohag 82524, Egypt; 3 Faculty of Biotechnology, Chemistry and Environmental Engineering, Phenikaa University, Hanoi 10000, Vietnam

**Keywords:** biphosphates, methanol electrooxidation, electrocatalysis, sol–gel, Fe/Ni

## Abstract

In this work, novel phosphate materials based on bimetallic character (Fe and Ni) were introduced by different chemical fabrication methods, the reflux method (FeNiP-R) and the sol–gel technique (FeNiP-S), and evaluated as non-precious electrodes for methanol electrooxidation in KOH electrolytes. The designed FeNiP-R and FeNiP-S samples were investigated using different characterization techniques, namely TEM, SEM, XPS, BET, DLS, and FT-IR, to describe the impact of the fabrication technique on the chemistry, morphology, and surface area. The characterization techniques indicate the successful fabrication of nanoscale-sized particles with higher agglomeration by the sol–gel technique compared with the reflux strategy. After that, the electrochemical efficiency of the fabricated FeNiP-R and FeNiP-S as electrodes for electrocatalytic methanol oxidation was studied through cyclic voltammetry (CV) at different methanol concentrations and scan rates in addition to impedance analysis and chronoamperometric techniques. From electrochemical analyses, a sharp improvement in the obtained current values was observed in both electrodes, FeNiP-R and FeNiP-S. During the MeOH electrooxidation over FeNiP-S, the current value was improved from 0.14 mA/cm^2^ at 0.402 V to 2.67 mA/cm^2^ at 0.619 V, which is around 109 times the current density value (0.0243 mA/cm^2^ at 0.62 V) found in the absence of MeOH. The designed FeNiP-R electrode showed an improved electrocatalytic character compared with FeNiP-S at different methanol concentrations up to 80 mmol/L. The enhancement of the anodic current density and charge transfer resistance indicates the methanol electrooxidation over the designed bimetallic Fe/Ni-phosphates.

## 1. Introduction

At the lab scale, the reported high-performance materials based on Pt or Pd metals are attractive candidates for electrochemical cells to produce energy from the stored chemical energy of renewable fuels, including ethanol [1], methanol [2,3], and formic acid [4]. However, the expected commercial cost and scarcity of these precious metals restrict their industrial applications on a large scale. Enormous efforts have been carried out for the electrode design of cost-effective, electrochemically stable electrochemical cells [5]. Recently, several electrodes based on non-precious materials have been reported for electrochemical fuel cells, such as 3D Hierarchical Graphitic C–Co NPs [6], polyaniline–Cu composites [7], CoCr-C nanofibers [8], Ni-ferrites [9], and Ni-phosphates [10]. Mono phosphates were studied to design effective electrodes having desired cost and performance [11,12]. For instance, nickel phosphate (Ni-P) was reported as an electrode material for formaldehyde oxidation [13], oxygen evolution reaction (OER) [14], hydrogen evolution reaction (HER) [15], water splitting [16,17], oxidation of glucose [18], urea oxidation [12,19], and methanol oxidation [10]. From these Ni-P materials, biphosphates were also studied as electrodes in electrochemical cells [13,16,17,20,21]. Ni was united with Co, Mn, Cr, or Fe to improve electrocatalytic behavior and stability. Thus, the chemical design of biphosphates is an attractive strategy in electrocatalysis to introduce non-precious and high-efficiency electrode materials.

With the updated technology and depletion of fossil fuels, environmental issues and energy overuse are considered among the most significant global issues. Electrochemical methods for the production of hydrogen fuels and electrical energy such as fuel cells are efficient and clean methods [22]. Among the different reported fuel cells, fuel cells based on methanol are the best suited for commercialization because methanol can provide some advantages as a utilized fuel, such as easy handling, transportation, and storage, being miscible with aqueous electrolytes, along with renewability and expected high power with high chemical rates, as methanol fuel lacks C-C bonds, and the cell could be started at low temperatures compared with other fuel cells [2,3,23]. However, the manufacturing cost still needs more research, and is related to the usage of Pt-based electrodes for the reduction of oxygen in the cathodic part and methanol electrooxidation in the anodic part [24]. The development of non-precious or Pt-free electrocatalysts for methanol electrooxidation is thus a key point to overcome the cost issue in fuel cells based on methanol. Among the reported metal electrocatalysts, nickel-based electrodes are potential electrodes due to their strong electrochemical activity through surface oxidation [8,11,25,26].

Compared to the reported noble metals such as Pt, other metals including Fe, Cu, and Ni, in addition to not having the carbon monoxide poisoning issue, are low-cost and exist in great quantities. Many researchers reported that these metals have acceptable and desired electrocatalytic efficiency towards fuel oxidation [27,28,29,30]. Although many studies reported that nickel-based electrodes have the desired electrocatalytic activity in many chemical applications, it could be improved by combination with iron [31,32]. Nickel has been combined with other metals including Pd [25], Co [26], Cr [8], Pt [33], Fe [34], and Sn [23] in order to enhance the electrocatalytic behavior. Compared to the cost of most transition metals, nickel is relatively inexpensive and electrochemically active and could be used for alcohol (including methanol and ethanol) electrooxidation, especially if incorporated with other metals.

Regarding Ni-based phosphate materials, it has HPO_4_^2−^ or PO_4_^3−^, which could improve the charge transfer during the electrooxidation process [35]. The Ni-alloys phosphate materials were reported as outstanding electrocatalysts in different electrochemical applications such as Fe/Ni-P for oxygen evolution reaction [36], water splitting [37], hydrogen evolution in alkaline or acidic media [38], and electrooxidation of methanol [39]. Ni_3_(PO_4_)_2_ working electrode was applied for electrooxidation of glucose [40], alcohol [11,41], and formaldehyde [42] in addition to overall water electrolysis [43]. Additionally, Fe-doped Ni-P material was reported as a synergistic electrocatalyst for water oxidation [44] as well as an electroactive catalyst for H_2_ evolution reaction [45]. Three-dimensional hierarchical Ni-P nanosheet arrays exhibit superior electrochemical capacitance [46]. Therefore, Ni-P could be considered a promising material to design working electrodes because of its easy design, low cost, and acceptable electrical conductivity in addition to significant faradaic pseudo capacitance [11]. The bimetallic character of Ni-based phosphate compounds could improve the electrochemical characteristics by enhancement of the Ni sites’ response to form the activated NiOOH [26].

In this work, bimetallic Fe/Ni-biphosphates were designed by two different chemical methods, sol–gel (FeNiP-S) and reflux (FeNiP-R), and investigated as electrode materials for methanol oxidation in KOH electrolytes. The impact of synthetic methods on morphology, chemistry, and surface area was studied via SEM, XPS, and BET. Furthermore, the electrocatalytic behavior of the fabricated FeNiP-S and FeNiP-R was extensively studied via cyclic voltammetry (CV) at different scan rates and fuel contents, along with chronoamperometric investigations and electrochemical impedance spectroscopy (EIS). The fabricated FeNiP-R material has an improved electrocatalytic performance for methanol oxidation.

## 2. Materials and Methods

### 2.1. Chemicals and Solutions

The utilized chemicals in this study are nickel (II) nitrate hexahydrate, iron (III) nitrate nonahydrate (≥99.95%), hexadecylpyridinium chloride surfactant, ammonium mono hydrogen phosphate (NH_4_)_2_HPO_4_, urea, and nitric acid (ACS reagent, 70%, Barcelona, Spain). All reagents were of analytical grade and were supplied by Sigma-Aldrich (Darmstadt, Germany). The used chemicals were pure and utilized as purchased, devoid of further purification.

### 2.2. Chemical Fabrication of FeNiP-R and FeNiP-S Materials

#### 2.2.1. Fabrication of FeNiP Based on Reflux Methodology (FeNiP-R)

The metallic (Fe and Ni) precursors were dissolved in water and refluxed at 90 °C for 6 h with concentrated nitric acid and urea aqueous solution, resulting in the formation of phosphates. The obtained blend was filtered, washed, and dried overnight. The obtained phosphate sample by reflux was coded as FeNiP-R.

#### 2.2.2. Fabrication of FeNiP Based on Sol–Gel Strategy (FeNiP-S)

The metallic precursors of Ni and Fe were dissolved in a mixture of double-distilled water/absolute ethanol (100:50 *v*/*v*%), keeping the Fe/Ni molar ratios as 1:1. Then, 1.8 g of HDPCl surfactant was dissolved well in 10 mL of deionized H_2_O and absolute ethanol. After, the surfactant solution was added dropwise to the Fe/Ni precursor solution, followed by the gradual addition of 1.0 M of urea solution, and stirring was continued for another 15 min. Urea was used as a capping agent, which stabilized the formation of small-size particles by inhibiting the over-growth of the formed particles or decreasing the rate of growth in addition to decreasing aggregation during material synthesis. The capping agent could stabilize the interface where nanoparticles interact with their parent solution (preparation medium). Then, the formed sol blend was added dropwise to 0.2 mol/L of ammonium phosphate and stirred well for 15 min. The pH of the obtained solution was fixed at around 6.5–7.5 using ammonia solution and stirred for 2.5 h at 40 °C. Then, the formed sol phosphate was sonicated for 0.5 h and aged overnight. The resulting precipitate was filtrated and washed by H_2_O and alcohol to remove any further adsorbed impurities, then dried. The formed Fe/Ni-phosphate was coded as FeNiP-S.

### 2.3. Physicochemical Characterization

The morphology of the designed FeNiP-R and FeNiP-S samples was investigated using a scanning electron microscope (FE-SEM/EDX, JSM-5410-Model-JEOL, Tokyo, Japan) and a transmission electron microscope (TEM) in a Jeol-1230 electron microscope operating at 200 KeV. The BET surface area of the fabricated FeNiP-R and FeNiP-S samples was derived from nitrogen adsorption–desorption measurements (Micro metrics ASAP2010) using the Brunauer–Emmett–Teller (BET) method. The FeNiP-R and FeNiP-S samples were degassed at 313 K before measurement in the degas pot of the adsorption analyzer. FTIR characterization was carried out on the BRUKER FTIR spectrometer in the range of 400–4000 cm^−1^.

### 2.4. Electrochemical Investigation

Electrochemical analysis including CV, CA, and EIS experiments were carried out using the Gamry instrument Potentiostat/Galvanostat/ZRA. The Gamry instrument includes the Echem software package, version ES260 Analyst 6.0 for data fitting and EIS-300 software for EIS measurements (Warminster, USA). The analyses were accomplished via the 3-electrode cell as reported before and by the use of common deposition at GCE [8,47]. The design of the working electrode was realized according to the previous study [9,10,48]. Pt-sheet and Ag/AgCl were utilized as auxiliary or counter and reference electrodes, respectively. The alkaline electrolyte was prepared by dissolving the solid KOH or methanol mix using bi-distilled ionized water to investigate the CA, CV, and EIS analyses at various methanol contents from 2 mM to 80 mM and at different scan rates. Cyclic voltammetry experiments were recorded from -0.2 V to 0.6 V. EIS experiments were examined in the frequency range from 0.1 Hz to 100 KHz.

## 3. Results and Discussion

### 3.1. Morphological and Chemical Analyses

**Figure 1:**
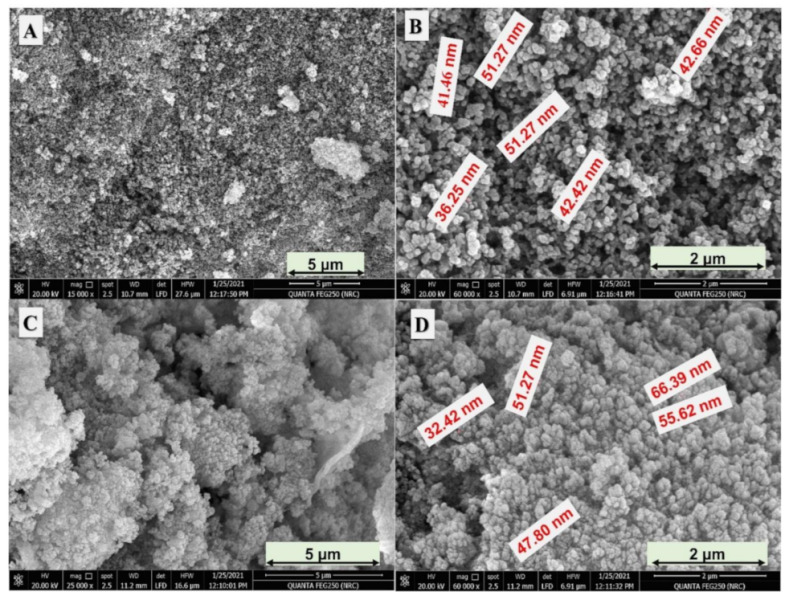
SEM images of the fabricated FeNiP-R at two different magnifications (**A**,**B**); FeNiP-S material at different magnifications (**C**,**D**).

**Figure 2:**
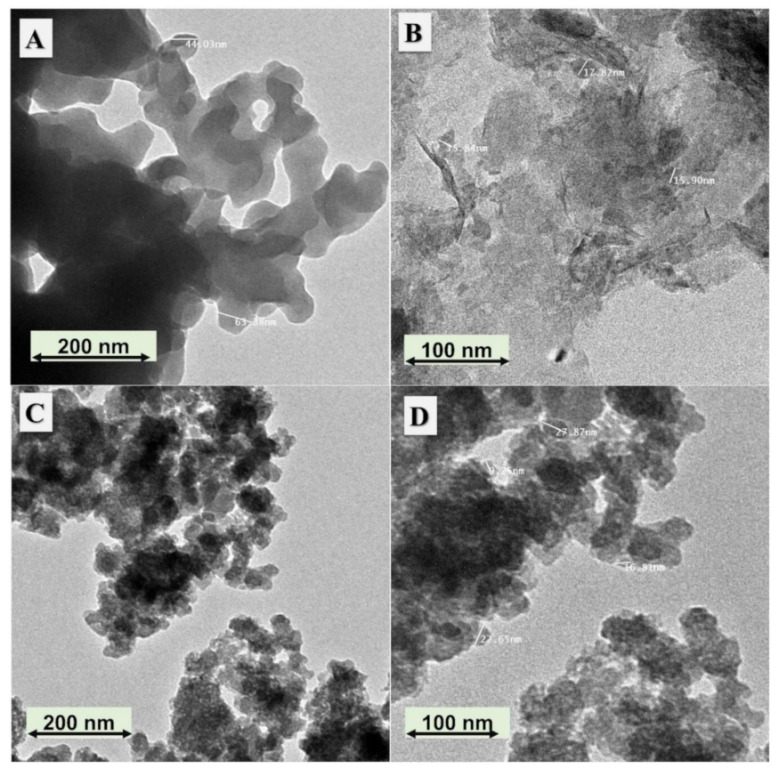
TEM images of the introduced FeNiP-R at two different magnifications (**A**,**B**); FeNiP-S material at different magnifications (**C**,**D**).

In this study, Fe and Ni bimetallic phosphate (FeNiP) was chemically fabricated by different preparation strategies, namely reflux (FeNiP-R) and sol–gel (FeNiP-S), to study this biphosphates material as a working electrode for methanol electrooxidation in alkaline medium for methanol fuel cells. The morphological analysis of the designed FeNiP-R and FeNiP-S was studied via SEM and TEM analyses. Figure 1A,B shows the SEM images of the FeNiP-R at different magnifications (Figure 1A at 15 KX and 1B at 60 KX). The SEM images exhibit nano-scale particles with size around 40 nm. The scale size of the formed phosphate particles was found to be between 35.25 nm and 51.27 nm, as shown in Figure 1B. In the case of FeNiP-S material (Figure 1C,D), the formed nanoparticles (NPs) have more aggregation compared with FeNiP-R NPs. The size of the formed FeNiP-S NPs was in the same range of the prepared FeNiP-R and found between 32.42 nm and 66.39 nm. The NPs of FeNiP-S materials are aggregated, which could be due to the decrease in the surface free energy during the sol–gel step, which will increase the bonding between nanoparticles and adhesion between the formed FeNiP-S NPs. The morphology character of the prepared FeNiP-R was confirmed through TEM, as displayed in Figure 2A,B. The TEM images of the FeNiP-R display particles connected together and mostly particles within nano-scale size. The FeNiP-S material was analyzed by TEM, as shown in Figure 2C,D. The aggregation difference in the morphology character between the prepared FeNiP-S and FeNiP-R could be attributed to the synthetic methodology and affirmed in the TEM images in addition to SEM images. The selected area diffraction (SAED) image of the FeNiP-S was supported, as displayed in Figure S1 (Supplementary Materials). The SAED shows gaps between lattice fringes, which indicate the monoclinic FeNiP phase.

**Figure 3:**
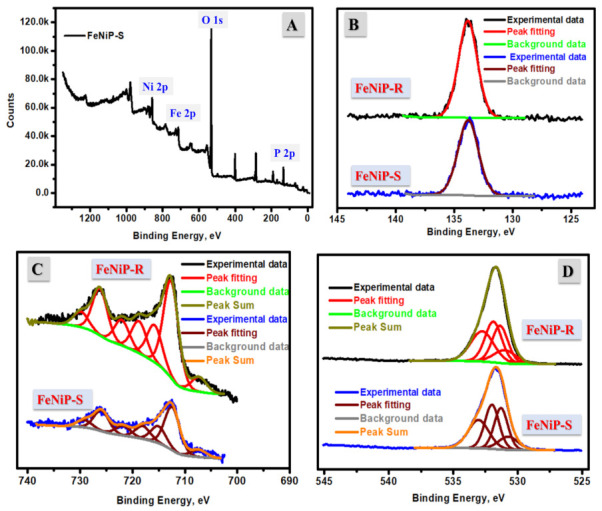
XPS analysis of the prepared FeNiP-S (**A**); fine XPS analysis of P-2p region (**B**); fine XPS analysis of Fe-2p region (**C**); fine XPS analysis of O-1s region (**D**).

The surface chemistry of the designed FeNiP-R and FeNiP-S was studied by XPS analysis, and the found data are displayed in Figure 3 and Table 1. The XPS survey scans of FeNiP-S and FeNiP-R are presented in Figure 3A and Figure S2 (Supplementary Materials), respectively, which indicated the presence of Fe 2p, O 1s, Ni 2p, and P 2p at 713.92 eV, 532.78 eV, 857.82 eV, and 134.73 eV, respectively. For FeNiP-S, the peaks of Fe, O, Ni, and P were found at 714.08 eV, 532.96 eV, 858.10 eV, and 134.82 eV, respectively. The difference in surface chemistry of the prepared FeNiP-R and FeNiP-S samples is nearly the same, which is due to the similar chemical content and the change in focus on the preparation strategy. The fine spectra XPS at a low scan rate of the prepared FeNiP-R and FeNiP-S samples in the binding energy range of P-2p, Fe-2p, O-1s, and Ni-2p are displayed in Figure 3B–D, and Figure S3 (Supplementary Materials), respectively. Figure 3B displays the P-2p core-level peak at 133.83 eV and 133.81 eV for the designed FeNiP-R and FeNiP-S, which could be due to the (PO_4_)^3−^ group [49]. The presence of only one peak indicates the presence of only one type of phosphate. The chemistry of phosphates in both FeNiP-R and FeNiP-S samples is similar, which indicates the successful preparation of phosphates by both preparation methods, reflux and sol–gel. In the XPS spectrum of Fe-2p (Figure 3C), the found peaks at binding energies 707.29 eV (Fe 2p_3/2_) and 722.05 eV (Fe 2p_1/2_) correspond to ferrous ions and the observed peaks at high binding energies 712.65 eV (Fe 2p_3/2_) and 726.22 eV (Fe 2p_1/2_) are attributed to the presence of ferric in FeNiP-R, which presents two spin–orbit doublets in the prepared FeNiP-R material [50,51]. In the case of FeNiP-S material, the ferrous peaks were found at binding energies 707.33 eV (Fe 2p_3/2_) and 721.80 eV (Fe 2p_1/2_) and the ferric peaks at binding energies 712.43 eV (Fe 2p_3/2_) and 725.93 eV (Fe 2p_1/2_), which present the existence of a ferrous ferric mixture in the FeNiP-S. The mixed valence state of iron metal in the FeNiP-R and FeNiP-S structure could enhance the oxidase catalytic behavior by enhancement of the electron transfer pathways [50,52]. The O 1s XPS spectra shown in Figure 3D affirms the presence of P-O bonds. The deconvoluted peaks in the range of the O 1s spectrum seen at 531.36 eV and 532.75 eV are indexed to P−OH and P−O, respectively [20,53]. For Ni XPS analysis, there are two main peaks observed at 861.83 eV and 857.82 eV in the Ni 2p, corresponding to Ni 2p_1/2_ and Ni 2p_3/2_, respectively (Figure S3, Supplementary Materials) [21]. Other satellite peaks were also seen at 874.47 eV and 865.34 eV around the previously mentioned Ni 2p peaks. All these peaks indicate the existence of Ni as Ni^2+^ in the prepared FeNiP materials.

### 3.2. Surface Area, DLS, FT-IR, XRD Analyses

**Figure 4:**
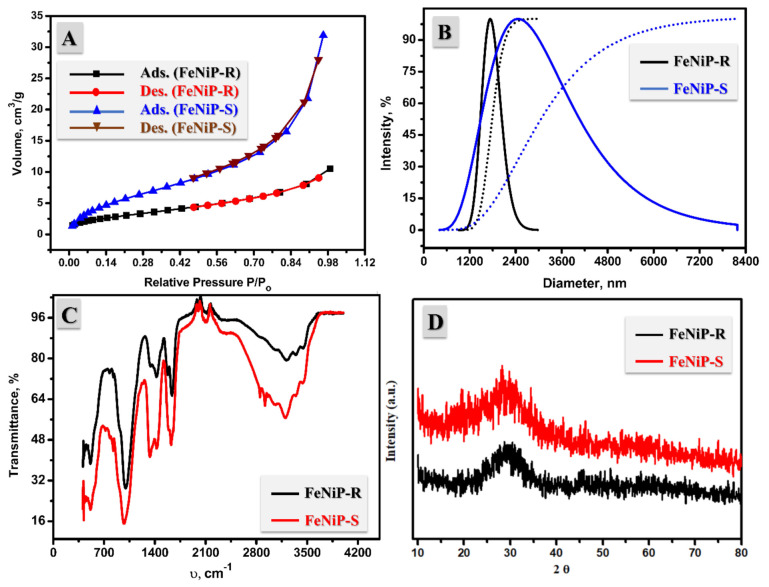
Nitrogen adsorption/desorption isotherm of FeNiP-R and FeNiP-S (**A**) and their DLS analysis (**B**), FT-IR spectroscopy (**C**), and XRD analysis (**D**).

The BET surface area analysis of the designed FeNiP-R and FeNiP-S biphosphate materials was performed through nitrogen adsorption (ads.)/desorption (des.) isotherm at 77 K, as displayed in Figure 4A. The N_2_-adsorption/desorption. curves of the prepared FeNiP-R and FeNiP-S samples coincide with type (IV) behavior, suggesting the presence of meso- and macropores [54]. The BET surface area of FeNiP-R and FeNiP-S samples was estimated and found to be 11.14 m2/g and 23.52 m^2^/g. Furthermore, the pore volume of FeNiP-R and FeNiP-S samples was found at 0.016 cm^3^/g and 0.049 cm^3^/g, and 7.72 m^2^/g. Therefore, the FeNiP-S sample has a better pore volume and surface area than the FeNiP-R sample, which could be due to the synthetic methodology, as the sol–gel technique allows the mesopores to form more easily than the reflux method. After that, the prepared FeNiP-R and FeNiP-S samples were investigated by dynamic light scattering (DLS) to describe and analyze particle scale size and provide the particle size distribution of the prepared materials. The observed particle size by DLS is larger than that previously seen via TEM, which could be attributed to the nature of DLS analysis, which describes the hydrodynamic diameter. This hydrodynamic diameter comprises the particles size and the electric dipole that sticks to the particle’s surface. The DLS analyses of FeNiP-R and FeNiP-S materials are shown in Figure 4B. The most common particle sizes were 1734.28 nm and 2455.04 nm for FeNiP-R and FeNiP-S, respectively. The larger particle size was found in the case of FeNiP-S compared to FeNiP-R. This could be attributed to the aggregation of particles in FeNiP-S, which was confirmed by SEM and TEM analyses (Figure 1 and Figure 2, respectively).

The FT-IR analysis of the prepared FeNiP-R and FeNiP-S samples was carried out and is organized in Figure 4C. IR spectra were utilized to indicate the function groups or chemical bonds, and the found FT-IR has broad peaks of the O-H bond at 3216.69 cm^−1^ and 3214.83 cm^−1^ for FeNiP-R and FeNiP-S samples, respectively, attributed to the crystallized water of metal phosphate, as reported before [55]. The broad peaks at 1332.52 cm^−1^ and 1433.16 cm^−1^ indicate the chemical bonds of the P = O bond of the PO_4_^3−^ function group in the case of FeNiP-R. For FeNiP-S, the vibrations of the PO_4_^3−^ function group were found at 1338.11 cm^−1^ and 1407.07 cm^−1^. Moreover, the clear phosphate peaks at 969.108 cm^−1^ and 1010.11 cm^−1^ for FeNiP-R and FeNiP-S, respectively, were ascribed to P-O stretching [56,57]. By another two bands in the 400–700 cm^−1^ range, it was confirmed that both FeNiP-R and FeNiP-S samples have oxide bonds (M(Fe or Ni)−O) plus an anionic PO_4_^3−^ group [58]. The FT-IR spectra indicate the existence of ^−^OH, P-O, P = O, and M (Fe or Ni)-P for both FeNiP-R and FeNiP-S samples, affirming the successful preparation of M-phosphate by both chemical methods, reflux and sol–gel. Additionally, XRD analysis is introduced in Figure 4D to describe the crystallinity of the prepared FeNiP-R and FeNiP-S. The XRD analysis shows that the prepared phosphate materials have an amorphous character with no clear sharp or crystalline peaks. The amorphous character of Fe/Ni-phosphate was reported before [36]. The prepared Fe/Ni-phosphate materials are amorphous because no detectable crystal structure could be seen in XRD. Amorphous phosphate materials could be prepared when there is not enough mobility to form crystalline structures. In short, the physicochemical characterizations indicate the successful synthesis of amorphous FeNi-P by reflux and sol–gel.

### 3.3. Electrochemical Analyses

The electrochemical analyses of simple fuels including methanol through novel low-cost materials as electrodes or anodes were studied by the use of Fe/Ni-biphosphates as the electrode material for renewable fuel cell devices. The electrochemical analyses including cyclic voltammetry, impedance studies, and chronoamperometric investigations were studied over Fe/Ni-biphosphates prepared by two simple methods, as discussed before.

#### 3.3.1. Cyclic Voltammetry (CV) Studies

**Figure 5:**
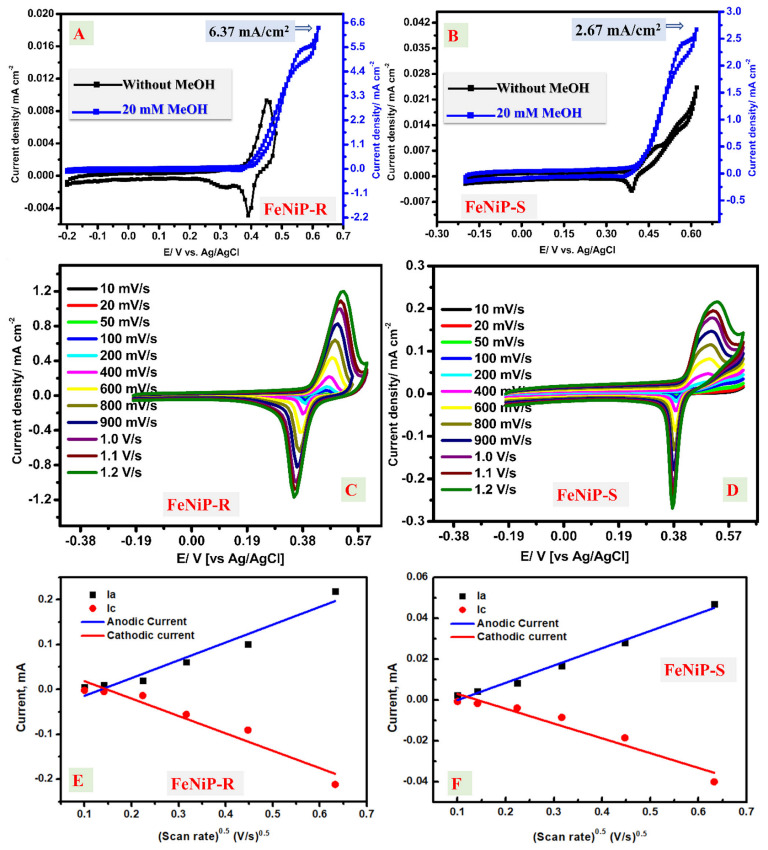
(**A**) Cyclic voltammograms of the FeNiP-R working electrode in KOH medium with and without 20 mM methanol. (**B**) Cyclic voltammograms of the FeNiP-S working electrode in KOH medium with and without 20 mM methanol. (**C**) Cyclic voltammograms of the FeNiP-R working electrode at different scan rates from 10 mV/s to 1200 mV/s using KOH aqueous solution as the electrolyte. (**D**) Cyclic voltammograms of the FeNiP-S working electrode at different scan rates from 5 mV/s to 400 mV/s using KOH aqueous solution as the electrolyte. (**E**) The variation in the peak current (Ip) vs. square root of the scan rate of the FeNiP-R working electrode. (**F**) The variation in the peak current (Ip) vs. square root of the scan rate of the FeNiP-S working electrode.

The CV technique was studied at different fuel contents and scan rates in the presence of KOH alone and KOH/MeOH solution. Figure 5A,B describes the CV characteristics of FeNiP-R and FeNiP-S GCE electrodes in KOH electrolytes with and without active fuel, 20 mM MeOH. For CV without MeOH, the peaks of reduction and oxidation could be easily observed at a voltage of 450 mV and 390 mV for the cathodic and anodic parts, respectively, for the sample of FeNiP-R (Figure 5A), and at 480 mV and 390 mV for the reduction and oxidation parts, respectively, for the sample of FeNiP-S (Figure 5B). These redox parts are traditionally expected in the characteristics of CV analysis when the working electrode material has Ni content, as in this study on FeNiP-R or FeNiP-S, and could be related to the reversible transformation of Ni ions, Ni ^(II)^/Ni^(III)^. The oxidation part was indicated by increasing the current density, which could be attributed to the electrochemical formation NiOOH from nickel hydroxide, and the reduction character is attributed to the formation of nickel hydroxide [25,59]. Interestingly, the oxidation part characteristics in the studied CV analysis in the case of 20 mM MeOH has an interesting increase in current values from 0.187 mA/cm^2^ at 0.409 V to 5.12 mA/cm^2^ at 0.549 V, which is around 59 times the highest current density value found in the absence of MeOH (0.0093 mA/cm^2^ at 0.45 V). The current density improvement confirms the interesting performance of the electrodes based on FeNiP-R for MeOH electrooxidation in high-pH environments. The observed electrooxidation of MeOH begins at 0.409 V vs. Ag/AgCl and 0.402 V for FeNiP-R and FeNiP-S, respectively. After that, a sharp improvement in current values was observed in both electrodes, FeNiP-R and FeNiP-S. During the MeOH oxidation over FeNiP-S, the current value was improved from 0.14 mA/cm^2^ at 0.402 V to 2.67 mA/cm^2^ at 0.619 V, which is around 109 times the current density value (0.0243 mA/cm^2^ at 0.62 V) found in the absence of MeOH. The CV analyses were repeated at a higher scan rate of 100 mV/s for FeNiP-R and FeNiP-S, as shown in Figures S4 and S5, respectively. The same trends and conclusions were found, and there was no considerable difference between what was found by high and low scan rates. The electrooxidation mechanism of MeOH has two parts: oxyhydroxide formation (OOH) and bond breakage to electrons [25,60,61]. The current density values increase because of MeOH oxidation over the synthesized FeNiP-R and FeNiP-S electrodes; then, after reaching the highest point, the current density started to stabilize. Therefore, the CV should be carried out in only KOH as alkaline media, which is discussed later.

The catalytic MeOH electrooxidation over the prepared FeNiP-R and FeNiP-S electrodes starts with Ni oxidation from +2 to +3 states as oxyhydroxide. Thus, the electrochemical activation of the prepared working electrodes was studied in only KOH media and is shown in Figure 5C,D for FeNiP-R and FeNiP-S, respectively. There are clear oxidation and reduction peaks of Ni at the voltages of 0.47 V and 0.38 V, respectively, in the case of FeNiP-R (Figure 5C). This redox character appeared because of the Ni +2 and +3 transformations. As the applied rate from 10 mV/s to 1.2 V/s increased, the current density values were enhanced in cathodic and anodic characteristics for both FeNiP-R and FeNiP-S cells. The Ni electrochemical activation in KOH electrolytes was executed in steps and initiated by the production of traditional nickel (II) hydroxide from Ni positions [62,63]. The nickel (II) hydroxide crystallinity has two forms, α-form and β-form. The latter is more stable and could be converted to β-NiOOH, which would be γ-NiOOH, and easily gathered in the KOH electrolyte at the active sites [64]. Figure 5 shows the Randles–Sevcik relation and Figure 5E displays the variation in the peak current (Ip) vs. square root of the scan rate based on the FeNiP-R working electrode [65,66]. Additionally, Figure 5F displays the variation in the peak current (Ip) vs. square root of the scan rate based on the FeNiP-S working electrode. The linear fit was applied for both figures, as represented inside the figure, and the R2 was found at 0.9735 and 0.9834 for FeNiP-R and FeNiP-S, respectively. From the slopes of the fitted straight lines of FeNiP-R (Figure 5E) and FeNiP-S (Figure 5F), the diffusion coefficient in cm^2^/s could be estimated and was found at 7.15 × 10^−6^ and 1.53 × 10^−6^ cm^2^/s for FeNiP-R and FeNiP-S, respectively. In short, the improvement of current density values at the anodic/cathodic peaks with the scan rate and the linear fitting indicate that the rate-determining step (RDS) for the presented FeNiP-R and FeNi-S of Ni-alkaline activation has diffusion behavior.

**Figure 6:**
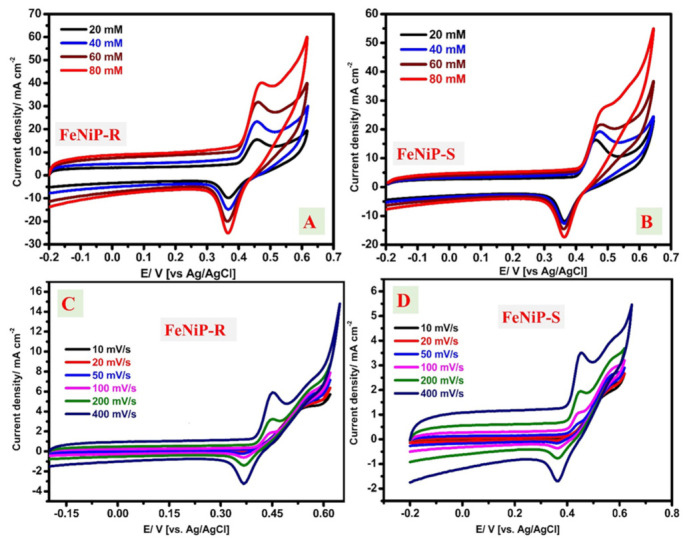
Cyclic voltammograms of FeNiP-R (**A**) and FeNiP-S (**B**) at different methanol concentrations from 20 mM to 80 mM. Cyclic voltammograms of the FeNiP-R working electrode (**C**) and the FeNiP-S working electrode (**D**) at different scan rates from 10 mV/s to 400 mV/s using 5 mM methanol in KOH aqueous solution as the electrolyte.

The commercial application of methanol fuel cells could be improved by the utilized fuel volume and thus the concentration of methanol. As the methanol concentration rises, the utilized cell volume could decrease and be easier to use. Using small cells could provide an advantage as it will be simply transported or stored. Previously, the electrical power increased from 10.0 to 20.0 mW.cm^−2^ because of only higher content from methanol [67]. Therefore, the MeOH content factor was investigated by CV at different MeOH concentrations from 20 mM to 80 mM for both FeNiP-R and FeNiP-S electrodes. Figure 6A and Figure 6B show the CV of the introduced FeNiP-R and FeNiP-S working electrodes at various MeOH concentrations, e.g., 20 mM, 40 mM, 60 mM, and 80 mM. The found peak values are organized in Table 2. The optimum methanol content was found at 80 mM according to the data obtained for both FeNiP-R and FeNiP-S electrodes. The methanol electrocatalytic oxidation is significantly enhanced with more methanol content at all investigated contents from 20 mM to 80 mM in the KOH electrolyte. The peak of FeNiP-R was detected at 0.458 V for 20 mM methanol and slightly increased to 0.472 V for higher methanol content of 80 mM. This small shift in the voltage of the oxidation peak could be due to the relative decrease in the hydroxide’s percentage at the electrode. The methanol electrochemical oxidation peak of FeNiP-S was observed at 0.465 V for 20 mM methanol and increased to 0.486 V for the high methanol concentration of 80 mM. Both FeNiP-R and FeNiP-S electrodes have an interesting performance for methanol electrooxidation, especially at 80 mM methanol, and FeNiP-R has a better performance than FeNiP-S, which was observed by the better current density values and lower required voltage values. Because of their electrochemical importance in the evaluation of the novel electrode materials, the electrochemically active surface area (ECSA) values of FeNiP-R and FeNiP-S electrodes were estimated from the cyclic voltammetric analysis according to the previous electrocatalysis study [68]. The ESCA values of the prepared FeNiP-R and FeNiP-S were found at 20.65 and 12.24 cm^2^. The higher ESCA for FeNiP-R over another phosphate material (FeNiP-S) confirms the superior electrocatalytic oxidation of methanol over FeNiP-R than FeNiP-S, in accordance with the direct peak current densities for the same methanol content.

The CV measurements were studied at different rates from 10 mV/s to 400 mV/s for the designed FeNiP-R and FeNiP-S electrodes using 20 mM methanol and 1 M KOH as the applied electrolyte, displayed in Figure 6C and Figure 6D, respectively. The redox peaks were remarkably enhanced and became wider between the reduction and oxidation curves as the applied scan rate grew. The cathodic peak was observed only at a high scan rate (>50 mV/s) and a sharp increase in the anodic peak was detected, which indicates the successful electrochemical oxidation of methanol with enhanced electrochemical kinetics. The improvement of CV area confirms the diffusion behavior of the rate-determining step during methanol electrooxidation [59,69]. These data suggest that the designed working electrodes based on FeNiP-R and FeNiP-S have an interesting electrocatalytic methanol oxidation with better electrochemical character in the case of the designed FeNiP-R compared with FeNiP-S. Table 3 gives a comparison between some low-cost materials (free from precious metals such as Pt or Au) that were recently reported for methanol electrocatalytic oxidation in alkaline medium. From this table, it can be deduced that the prepared FeNiP-R and FeNiP-S are efficient electrocatalysts for methanol electrooxidation at high pH.

#### 3.3.2. Chronoamperometric (CA) Studies

**Figure 7:**
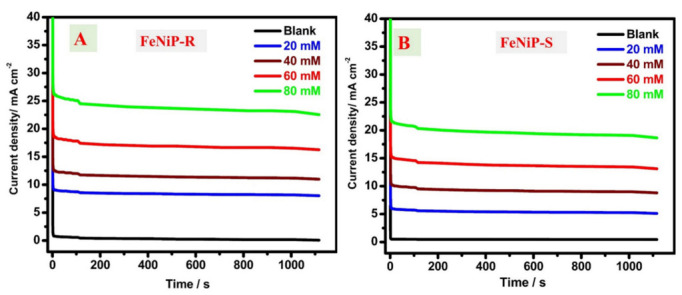
Chronoamperometric analysis in 0.0 M, 20 mM, 40 mM, 60 mM, and 80 mM methanol/KOH aqueous electrolyte of the prepared FeNiP-R; (**A**), and FeNiP-S; (**B**).

CA analyses were conducted for the designed FeNiP-R and FeNiP-S working electrodes at different MeOH contents (0.0 mM, 20 mM, 40 mM, and 80 mM), as presented in Figure 7A,B, respectively. The obtained current values of FeNiP-R and FeNiP-S working electrodes after 1016 s are given in Figure S6 (Supplementary Materials). The current density value after 1016 s increased as the investigated methanol concentration rose. For the FeNiP-R electrode, the current density was found at 8.173 mA/cm^2^ for 0.02 mol/L MeOH/KOH and 0.17 mA/cm^2^ for the 1.0 M KOH electrolyte without methanol. The current density of the FeNiP-S electrode was found at 5.26 mA/cm^2^ for 0.02 mol/l MeOH/KOH and 0.46 mA/cm^2^ for the 1.0 M KOH electrolyte without methanol. Hence, the anodic current was improved because of the methanol content, which indicates the significant electrocatalytic character of FeNiP-R and FeNiP-S electrodes for methanol electrooxidation. The deactivation rate of the obtained current could be calculated via CA data at 600 s and 60 s by the use of the reported equation: ((J_60s_− J_600s_)/J_60s_)) [25,76]. The deactivation rate was estimated as 6.16% and 7.61% for FeNiP-R and FeNiP-S working electrodes, respectively, indicating the high electrochemical stability of the designed electrodes, and the difference between them could be ignored (around 1.45%).

#### 3.3.3. Electrochemical Impedance Spectroscopic (EIS) Studies

**Figure 8:**
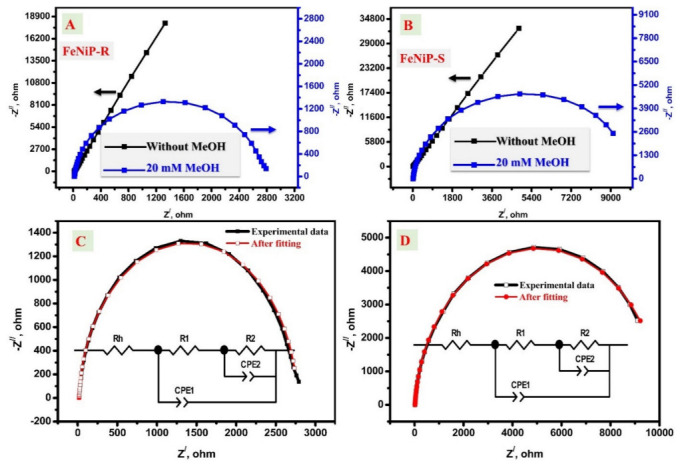
The Nyquist plots of the studied FeNiP-R (**A**) and FeNiP-S (**B**) in 0.0 M methanol and 20 mM urea in 1.0 M KOH. The difference between the fitted and experimental data of the prepared FeNiP-R (**C**) and FeNiP-S (**D**) electrodes.

**Figure 9:**
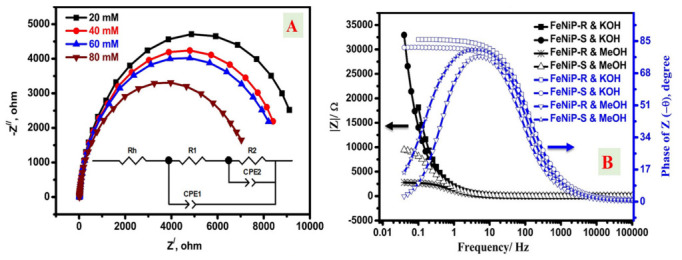
The Nyquist (Z^/^, -Z^//^) plots at different methanol concentrations from 20 mM to 80 mM methanol (**A**); total impedance and bode phase plots of the FeNiP-R and FeNiP-S electrodes in the presence and absence of methanol (**B**).

The EIS investigations of the designed FeNiP-R and FeNiP-S working electrodes in the absence and presence of 20 mM methanol in KOH solution are displayed in Figure 8A and Figure 8B, respectively. In the case of the electrolyte without methanol, the Nyquist plot is displayed as a line behavior without a full semi-circle, which indicates the low charge transfer process in the KOH electrolyte without methanol. The impedance imaginary part in the EIS curve without methanol was increased and nearly a full semi-circle line emerged, indicating that the fabricated biphosphates electrodes possess a capacitive behavior [77]. For 20 mM methanol/KOH electrolyte, a full semi-circle was observed, which confirms the presence of an additional charge transfer process (methanol electrooxidation) [48,78]. Generally, the low impedance Z^/^ and Z^//^ values indicate the presence of an efficient charge transfer process with better conductivity [61,79] for both investigated FeNiP-R and FeNiP-S electrodes and 20 mM methanol compared to electrolytes without methanol. In the experimental cell, there are two expected charge transfer processes besides the series/ohmic resistance for the alkaline solution, the first for the Ni-electrooxidation, and the second for the methanol oxidation. The charge transfer process is better in the case of methanol, which adopted the full semi-circle character in both FeNiP-R and FeNiP-S working electrodes. The resistance of charge transfer could be studied via the fitting analysis by the use of the presented equivalent circuit in the inset figure of Figure 8C [26,61,80]. The successful fitting was affirmed by the ignored difference between the pristine and fitted data, as shown in Figure 8C and 8D for FeNiP-R and FeNiP-S working electrodes, respectively. Moreover, the effect of MeOH contents on the EIS character was studied, as shown in Figure 9A, and the fitting parameters are organized in Table 4. The fitting parameters of the applied electrical circuit have two *R*_ct_ and one series *R*_h_. The series resistance (Rh) was slightly decreased with the increase in MeOH, which could be interpreted as the poor conductivity of MeOH compared with the KOH solution. The other components of equivalent circuit parameters (R_1_/CPE1 and R_2_/CPE2) are attributed to the oxidation processes, Ni oxidation and MeOH oxidation, respectively. Interestingly, both Rct resistances (R1 and R2) were decreased after methanol was introduced to the electrolyte. The value of R1 was changed from 10.63 ohm to 9.79 ohm after the change in methanol concentration from 0.0 to 20 mM, which affirms the superior Ni oxidation. For R2, the resistance was changed from 13,144 KΩ to 10.08 KΩ because of methanol, which indicates the existence of a novel charge transfer process originating from methanol electrooxidation. Figure 9B shows the total impedance behavior with bode phase character for FeNiP-R and FeNiP-S electrodes in KOH solutions with and without 20 mM methanol. The total impedance curves were shifted to lower values with methanol compared to curves without methanol for both evaluated electrodes, FeNiP-R and FeNiP-S. For bode phase plots, both curves have peak character with methanol, and no peaks could be detected in the bode phase without methanol, demonstrating the electrochemical oxidation of methanol by the investigated FeNiP-R and FeNiP-S electrodes. Generally, the charge transfer processes were improved in the presence of methanol, as confirmed by Nyquist plots, fitting analysis, bode phase, and total impedance data. Therefore, the introduced FeNiP-R and FeNiP-S working electrodes are suitable electrodes for methanol oxidation at high pH to design novel non-precious electrodes for methanol fuel cells.

## 4. Conclusions

Fe/Ni-biphosphates as novel electrocatalysts for methanol electrooxidation were fabricated by two chemical techniques (FeNiP-R by reflux, and FeNiP-S using sol–gel). The prepared FeNiP-R and FeNiP-S materials were characterized by SEM, XPS, TEM, FT-IR, and BET to analyze their morphology, surface area, and chemical bonds. The scale size of the formed phosphate particles was between 35.25 nm and 51.27 nm. In the case of FeNiP-S material, the formed nanoparticles (NPs) have more aggregation than FeNiP-R NPs. The electrochemical analysis of the prepared FeNiP-R and FeNiP-S materials as electrodes for electrocatalytic oxidation of methanol was inspected through CV as well as CA and EIS analyses to indicate the efficiency in terms of the obtained anodic current and charge transfer. These analyses affirm the high performance of the methanol electrooxidation over FeNiP-R and FeNiP-S electrodes. Interestingly, the obtained oxidation current improved as methanol concentration increased to 2 mM. From the CV analysis, the electrooxidation in the case of 20 mM MeOH had an interesting increase in current values from 0.187 mA/cm^2^ at 0.409 V to 5.12 mA/cm^2^ at 0.549 V, which is around 59 times the highest current density value found in the absence of MeOH (0.0093 mA/cm^2^ at 0.45 V). Notably, both series and charge transfer resistances decreased after methanol addition to the electrolyte. The value of series resistance was changed from 10.63 ohm to 9.79 ohm after the change in methanol concentration from 0.0 to 20 mM, which affirms the superior electrooxidation. The deactivation rates of the anodic current density were estimated as 6.16% and 7.61% for FeNiP-R and FeNiP-S working electrodes, respectively, indicating the high electrochemical stability of the designed FeNiP-R and FeNiP-S electrodes. This study presents novel non-precious Fe/Ni-biphosphates for the simple fabrication of electrodes in methanol fuel cells.

## Figures and Tables

**Table 1:** XPS analysis of the prepared FeNiP-S material.

Peak	Start BE	Peak BE	End BE	Height CPS	FWHM eV	Atomic %
O1s	539.08	532.96	523.08	95,796.76	3.41	44.35
Ni2p	871.08	858.1	843.08	16,208.02	4.62	22.18
P2p	140.58	134.82	129.08	11,595.97	3.22	1.192
Fe2p	736.08	714.08	702.08	11,659.48	6.66	16.42

**Table 2:** Current densities and their voltages of the FeNiP-R and FeNiP-S working electrodes at the anodic peak of CV analyses.

No.	Material	MeOH in KOH	Current Density, mA/cm^2^	Peak Potential, V
1	FeNiP-R	20 mM	15.60	0.458
2		40 mM	23.40	0.458
3		60 mM	31.80	0.458
4		80 mM	40.25	0.472
5	FeNiP-S	20 mM	16.40	0.465
6		40 mM	19.25	0.472
7		60 mM	21.65	0.479
8		80 mM	27.39	0.486

**Table 3:** Comparison of the activity of some reported electrocatalysts for methanol oxidation in alkaline media.

No.	Electrocatalyst Material	MeOH Concentration	Current Density, mA/cm^2^	Reference
1	Co/Cu @CNFs	2 M	17.5	[70]
2	Co/CeO_2_-decorated C fibers	3 M	9.5	[71]
3	ZnO/CeO_2_ dots@CNFs	3 M	16.3	[72]
4	Co/SrCO_3_ nanorods	3 M	10.0	[73]
5	Ni/Polypyrrole/RGO	1 M	32.94	[74]
6	Ni/PdCNFs	1 M	7.15	[75]
7	FeNiP-R	0.08 M	40.25	This work
8	FeNiP-S	0.08 M	27.39	

**Table 4:** Equivalent circuit parameters as derived from the fitted findings of the impedance data based on the FeNiP-S working electrode.

No.	Material	*R*_h_/Ω	*R*_1_/Ω	*R*_2_/kΩ	CPE1-T(+), µF	CPE1-P(+)/F	CPE2-T(+)/µF	CPE2-P(+)/F
1	0.0 mM	16.20	10.63	13144.00	59.16	0.92	49.59	0.90
Error, %		0.17	27.74	1.84	31.67	3.22	37.91	3.33
2	20.0 mM	16.71	9.79	10.08	50.08	0.96	53.81	0.94
Error, %		0.21	25.82	0.35	37.90	3.86	35.41	3.14
3	40 mM	16.32	9.56	9.10	50.35	0.96	53.96	0.94
Error, %		0.21	25.21	0.48	36.93	3.75	34.58	3.07
4	60 mM	17.16	9.17	8.69	49.09	0.96	53.87	0.94
Error, %		0.22	26.81	0.48	40.21	4.06	36.77	3.26
5	80 mM	18.09	8.95	7.19	45.13	0.96	55.25	0.94
Error, %		0.24	31.21	0.51	52.03	5.28	43.01	3.69

## Data Availability

The raw/processed data generated in this work are available upon request from the corresponding author.

## Supplementary Materials

The following supporting information can be downloaded at: https://www.mdpi.com/article/10.3390/nano12193429/s1, Figure S1: SAED of the synthesized FeNiP-R material; Figure S2: Total survey XPS of the prepared FeNiP-R material in the range of 0–1400 eV; Figure S3: XPS fine spectra of the fabricated FeNiP-R and FeNiP-S materials in the nickel region; Figure S4: Cyclic voltammograms of the FeNiP-R working electrode in KOH medium with and without 20 mM methanol at 100 mV/s; Figure S5: Cyclic voltammograms of the FeNiP-S working electrode in KOH medium with and without 20 mM methanol at 100 mV/s; Figure S6: The obtained current values of FeNiP-R and FeNiP-S working electrodes after 1016 s.

## References

[1] Huynh T.T., Dang N.N., Pham H.Q. (2021). Bimetallic PtIr nanoalloy on TiO2-based solid solution oxide with enhanced oxygen reduction and ethanol electro-oxidation performance in direct ethanol fuel cells. Catal. Sci. Technol..

[2] Yang Y., Tan C., Yang Y., Zhang L., Zhang B.W., Wu K.H., Zhao S. (2021). Pt3Co@ Pt core@ shell nanoparticles as efficient oxygen reduction electrocatalysts in direct methanol fuel cell. ChemCatChem.

[3] Zhao F., Zheng L., Yuan Q., Yang X., Zhang Q., Xu H., Guo Y., Yang S., Zhou Z., Gu L. (2021). Ultrathin PdAuBiTe Nanosheets as High-Performance Oxygen Reduction Catalysts for a Direct Methanol Fuel Cell Device. Adv. Mater..

[4] Li X., Zhang J., Jin C., Yan B., Cai J., Li M., Peng X., Wang Y. (2021). Tailoring Reaction Pathways by Tuning the Surface Composition of AuPt Nanocatalysts for Enhanced Formic Acid Oxidation. ACS Sustain. Chem. Eng..

[5] Sharma P., Minakshi Sundaram M., Watcharatharapong T., Laird D., Euchner H., Ahuja R. (2020). Zn metal atom doping on the surface plane of one-dimesional NiMoO4 nanorods with improved redox chemistry. ACS Appl. Mater. Interfaces.

[6] Alotaibi N., Hammud H.H., Al Otaibi N., Prakasam T. (2020). Electrocatalytic Properties of 3D Hierarchical Graphitic Carbon–Cobalt Nanoparticles for Urea Oxidation. ACS Omega.

[7] Sonawane J.M., Pant D., Ghosh P.C., Adeloju S.B. (2021). Polyaniline–Copper Composite: A Non-precious Metal Cathode Catalyst for Low-Temperature Fuel Cells. Energy Fuels.

[8] Mohamed I.M., Motlak M., Fouad H., Barakat N.A. (2016). Cobalt/chromium nanoparticles-incorporated carbon nanofibers as effective nonprecious catalyst for methanol electrooxidation in alkaline medium. Nano.

[9] Mohammed F.A., Khalaf M.M., Mohamed I.M.A., Saleh M.M., El-Lateef H.M.A. (2020). Synthesis of mesoporous nickel ferrite nanoparticles by use of citrate framework methodology and application for electrooxidation of glucose in alkaline media. Microchem. J..

[10] Khalaf M.M., Abd El-Lateef H.M., Touny A.H., Saleh M.M., Mohamed I.M.A. (2021). Electrocatalytic performance of inorganic nanoflakes nickel phosphates under adjusted synthetic parameters towards urea and methanol oxidation in alkaline media. Microchem. J..

[11] Kottayintavida R., Gopalan N.K. (2020). Nickel phosphate modified carbon supported Pd catalyst for enhanced alcohol electro oxidation. Int. J. Hydrogen Energy.

[12] Tsai Y.-C., Wu M.-S. (2020). Zeolitic nickel phosphate nanorods with open-framework structure (VSB-5) for catalytic application in electro-oxidation of urea. Appl. Surf. Sci..

[13] Zhou Z., Zeng L., Xiong G., Yang L., Yuan H., Yu J., Xu S., Wang D., Zhang X., Liu H. (2021). Multifunctional Electrocatalyst of NiCo-NiCoP Nanoparticles Embedded into P-doped Carbon Nanotubes for Energy-Saving Hydrogen Production and Upgraded Conversion of Formaldehyde. Chem. Eng. J..

[14] Liu H., Li H., Wang X. (2016). Electrostatic Interaction-Directed Growth of Nickel Phosphate Single-Walled Nanotubes for High Performance Oxygen Evolution Reaction Catalysts. Small.

[15] Kucernak A.R., Sundaram V.N.N. (2014). Nickel phosphide: The effect of phosphorus content on hydrogen evolution activity and corrosion resistance in acidic medium. J. Mater. Chem. A.

[16] Li C., Mei X., Lam F.L.-Y., Hu X. (2020). Hybridizing amorphous nickel cobalt phosphate and nickel phosphide as an efficient bifunctional nanocatalyst towards overall water splitting. Catal. Today.

[17] He L., Gong L., Gao M., Yang C.-W., Sheng G.-P. (2020). In situ formation of NiCoP@ phosphate nanocages as an efficient bifunctional electrocatalyst for overall water splitting. Electrochim. Acta.

[18] Pujari S.S., Kadam S.A., Ma Y.-R., Khalate S.A., Katkar P.K., Marje S.J., Patil U.M. (2021). Highly sensitive hydrothermally prepared nickel phosphate electrocatalyst as non-enzymatic glucose sensing electrode. J. Porous Mater..

[19] Li Q., Li X., Gu J., Li Y., Tian Z., Pang H. (2021). Porous rod-like Ni2P/Ni assemblies for enhanced urea electrooxidation. Nano Res..

[20] Chinnadurai D., Rajendiran R., Li O.L., Prabakar K. (2021). Mn-Co bimetallic phosphate on electrodeposited PANI nanowires with composition modulated structural morphology for efficient electrocatalytic water splitting. Appl. Catal. B Environ..

[21] Sun C., Sun L., Zhang Y., Si H., Fan K., Shi Y., Gu J., Zhang Y. (2020). Reduced graphene oxide-modified NiCo-phosphates on Ni foam enabling high areal capacitances for asymmetric supercapacitors. J. Mater. Sci. Technol..

[22] Cullen D.A., Neyerlin K.C., Ahluwalia R.K., Mukundan R., More K.L., Borup R.L., Weber A.Z., Myers D.J., Kusoglu A. (2021). New roads and challenges for fuel cells in heavy-duty transportation. Nat. Energy.

[23] Li J., Luo Z., Zuo Y., Liu J., Zhang T., Tang P., Arbiol J., Llorca J., Cabot A. (2018). NiSn bimetallic nanoparticles as stable electrocatalysts for methanol oxidation reaction. Appl. Catal. B Environ..

[24] Sgroi M.F., Zedde F., Barbera O., Stassi A., Sebastián D., Lufrano F., Baglio V., Aricò A.S., Bonde J.L., Schuster M. (2016). Cost analysis of direct methanol fuel cell stacks for mass production. Energies.

[25] Mohamed I.M.A., Yasin A.S., Barakat N.A.M., Song S.A., Lee H.E., Kim S.S. (2018). Electrocatalytic behavior of a nanocomposite of Ni/Pd supported by carbonized PVA nanofibers towards formic acid, ethanol and urea oxidation: A physicochemical and electro-analysis study. Appl. Surf. Sci..

[26] Mohamed I.M.A., Liu C. (2019). Chemical design of novel electrospun CoNi/Cr nanoparticles encapsulated in C-nanofibers as highly efficient material for urea oxidation in alkaline media. Appl. Surf. Sci..

[27] Fang Z., Zhang P., Wang M., Li F., Wu X., Fan K., Sun L. (2021). Selective Electro-oxidation of Alcohols to the Corresponding Aldehydes in Aqueous Solution via Cu (III) Intermediates from CuO Nanorods. ACS Sustain. Chem. Eng..

[28] Sreekanth T., Dillip G., Nagajyothi P., Yoo K., Kim J. (2021). Integration of Marigold 3D flower-like Ni-MOF self-assembled on MWCNTs via microwave irradiation for high-performance electrocatalytic alcohol oxidation and oxygen evolution reactions. Appl. Catal. B Environ..

[29] Liu B., Zhang M., Liu Y., Wang Y., Yan K. (2021). Electrodeposited 3D hierarchical NiFe microflowers assembled from nanosheets robust for the selective electrooxidation of furfuryl alcohol. Green Energy Environ..

[30] Shi Y., Li H., Ao D., Chang Y., Xu A., Jia M., Jia J. (2021). 3D nickel diselenide architecture on nitrogen-doped carbon as a highly efficient electrode for the electrooxidation of methanol and urea. J. Alloys Compd..

[31] Zhou Y., Yan D., Gu Q., Zhu S., Wang L., Peng H., Zhao Y. (2021). Implanting cation vacancies in Ni-Fe LDHs for efficient oxygen evolution reactions of lithium-oxygen batteries. Appl. Catal. B Environ..

[32] Li M., Li H., Jiang X., Jiang M., Zhan X., Fu G., Lee J.-M., Tang Y. (2021). Gd-induced electronic structure engineering of a NiFe-layered double hydroxide for efficient oxygen evolution. J. Mater. Chem. A.

[33] Li W., Jen Shih Y., Sanchez Carretero D., Huang C.-P. (2022). The electrochemical oxidation of chloride on Pt-Ni-Co-G electrodes and its application in in-situ disinfection of water. Chem. Eng. J..

[34] Barakat N.A.M., Shaheer Akhtar M., Mohamed I.M.A., Dakka Y.A., Hamdan R., El-Deen A.G., Elsaid K., Obaid M., Al-Meer S. (2017). Effective and stable FeNi@ N-doped graphene counter electrode for enhanced performance dye sensitized solar cells. Mater. Lett..

[35] Li J., Xu W., Zhou D., Luo J., Zhang D., Xu P., Wei L., Yuan D. (2018). Synthesis of 3D flower-like cobalt nickel phosphate grown on Ni foam as an excellent electrocatalyst for the oxygen evolution reaction. J. Mater. Sci..

[36] Ren H., Sun X., Du C., Zhao J., Liu D., Fang W., Kumar S., Chua R., Meng S., Kidkhunthod P. (2019). Amorphous Fe–Ni–P–B–O nanocages as efficient electrocatalysts for oxygen evolution reaction. ACS Nano.

[37] Guo M., Song S., Zhang S., Yan Y., Zhan K., Yang J., Zhao B. (2020). Fe-Doped Ni–Co Phosphide Nanoplates with Planar Defects as an Efficient Bifunctional Electrocatalyst for Overall Water Splitting. ACS Sustain. Chem. Eng..

[38] Song S., Guo M., Zhang S., Zhan K., Yan Y., Yang J., Zhao B., Xu M. (2020). Plasma-assisted synthesis of hierarchical NiCoxPy nanosheets as robust and stable electrocatalyst for hydrogen evolution reaction in both acidic and alkaline media. Electrochim. Acta.

[39] Chen S., Yang X., Tong X., Zhang F., Zou H., Qiao Y., Dong M., Wang J., Fan W. (2020). Design of 3D hollow porous heterogeneous nickel–cobalt phosphides for synergistically enhancing catalytic performance for electrooxidation of methanol. ACS Appl. Mater. Interfaces.

[40] Al-Omair M.A., Touny A.H., Al-Odail F.A., Saleh M.M. (2017). Electrocatalytic Oxidation of Glucose at Nickel Phosphate Nano/Micro Particles Modified Electrode. Electrocatalysis.

[41] Yang J., Tan J., Yang F., Li X., Liu X., Ma D. (2012). Electro-oxidation of methanol on mesoporous nickel phosphate modified GCE. Electrochem. Commun..

[42] Touny A.H., Tammam R.H., Saleh M.M. (2018). Electrocatalytic oxidation of formaldehyde on nanoporous nickel phosphate modified electrode. Appl. Catal. B Environ..

[43] Song D., Hong D., Kwon Y., Kim H., Shin J., Lee H.M., Cho E. (2020). Highly porous Ni–P electrode synthesized by an ultrafast electrodeposition process for efficient overall water electrolysis. J. Mater. Chem. A.

[44] Li Y., Zhao C. (2016). Iron-Doped Nickel Phosphate as Synergistic Electrocatalyst for Water Oxidation. Chem. Mater..

[45] Theerthagiri J., Cardoso E.S.F., Fortunato G.V., Casagrande G.A., Senthilkumar B., Madhavan J., Maia G. (2019). Highly Electroactive Ni Pyrophosphate/Pt Catalyst toward Hydrogen Evolution Reaction. ACS Appl. Mater. Interfaces.

[46] Liu Q., Chen C., Zheng J., Wang L., Yang Z., Yang W. (2017). 3D hierarchical Ni(PO3)2 nanosheet arrays with superior electrochemical capacitance behavior. J. Mater. Chem. A.

[47] Abd El-Lateef H.M., Khalaf M.M., Mohamed I.M.A. (2020). An efficient and non-precious anode electrocatalyst of NiO-modified carbon nanofibers towards electrochemical urea oxidation in alkaline media. Ceram. Int..

[48] Abd El-Lateef H.M., Almulhim N.F., Mohamed I.M.A. (2019). Physicochemical and electrochemical investigations of an electrodeposited CeNi2@NiO nanomaterial as a novel anode electrocatalyst material for urea oxidation in alkaline media. J. Mol. Liq..

[49] Chinnadurai D., Nallal M., Kim H.J., Li O.L., Park K.H., Prabakar K. (2020). Mn3+ Active Surface Site Enriched Manganese Phosphate Nano-polyhedrons for Enhanced Bifunctional Oxygen Electrocatalyst. ChemCatChem.

[50] Ojha R.P., Pal S., Prakash R. (2021). Cu-Fe Prussian blue analog nanocube with intrinsic oxidase mimetic behaviour for the non-invasive colorimetric detection of Isoniazid in human urine. Microchem. J..

[51] Xing X., Song Y., Jiang W., Zhang X. (2020). CuFe-P from a Prussian blue analogue as an electrocatalyst for efficient full water splitting. Sustain. Energy Fuels.

[52] Xuan C., Wang J., Xia W., Peng Z., Wu Z., Lei W., Xia K., Xin H.L., Wang D. (2017). Porous Structured Ni-Fe-P Nanocubes Derived from a Prussian Blue Analogue as an Electrocatalyst for Efficient Overall Water Splitting. ACS Appl. Mater. Interfaces.

[53] Zhang L., Liu Y., Wang Y., Li X., Wang Y. (2021). Investigation of phosphate removal mechanisms by a lanthanum hydroxide adsorbent using p-XRD, FTIR and XPS. Appl. Surf. Sci..

[54] Iqbal W., Yang B., Zhao X., Rauf M., Mohamed I.M.A., Zhang J., Mao Y. (2020). Facile one-pot synthesis of mesoporous g-C3N4 nanosheets with simultaneous iodine doping and N-vacancies for efficient visible-light-driven H2 evolution performance. Catal. Sci. Technol..

[55] Li J., Zhang L., Yang P., Cheng X. (2018). Morphological evolution of Co phosphate and its electrochemical and photocatalytic performance. CrystEngComm.

[56] Kim K.H., Jeong J.-M., Lee S.J., Choi B.G., Lee K.G. (2016). Protein-directed assembly of cobalt phosphate hybrid nanoflowers. J. Colloid Interface Sci..

[57] Arunachalam P., Shaddad M.N., Alamoudi A.S., Ghanem M.A., Al-Mayouf A.M. (2017). Microwave-Assisted Synthesis of Co3(PO4)2 Nanospheres for Electrocatalytic Oxidation of Methanol in Alkaline Media. Catalysts.

[58] Alsafrani A.E., Adeosun W.A., Alruwais R.S., Marwani H.M., Asiri A.M., Khan I., Khan A. (2022). Preparation, characterization and super electrocatalytic sensing study of polyaniline@yttrium phosphate (PANI@Y(III)PO4) nanocomposite. J. Mater. Res. Technol..

[59] Vedharathinam V., Botte G.G. (2012). Understanding the electro-catalytic oxidation mechanism of urea on nickel electrodes in alkaline medium. Electrochim. Acta.

[60] Barakat N.A.M., Yassin M.A., Al-Mubaddel F.S., Amen M.T. (2018). New electrooxidation characteristic for Ni-based electrodes for wide application in methanol fuel cells. Appl. Catal. A Gen..

[61] Mohamed I.M., Kanagaraj P., Yasin A.S., Iqbal W., Liu C. (2020). Electrochemical impedance investigation of urea oxidation in alkaline media based on electrospun nanofibers towards the technology of direct-urea fuel cells. J. Alloys Compd..

[62] Van der Ven A., Morgan D., Meng Y., Ceder G. (2006). Phase stability of nickel hydroxides and oxyhydroxides. J. Electrochem. Soc..

[63] Barakat N.A., El-Newehy M.H., Yasin A.S., Ghouri Z.K., Al-Deyab S.S. (2016). Ni&Mn nanoparticles-decorated carbon nanofibers as effective electrocatalyst for urea oxidation. Appl. Catal. A Gen..

[64] Barakat N.A., Motlak M., Elzatahry A.A., Khalil K.A., Abdelghani E.A. (2014). NixCo1−x alloy nanoparticle-doped carbon nanofibers as effective non-precious catalyst for ethanol oxidation. Int. J. Hydrogen Energy.

[65] Abdi Z., Vandichel M., Sologubenko A.S., Willinger M.-G., Shen J.-R., Allakhverdiev S.I., Najafpour M.M. (2021). The importance of identifying the true catalyst when using Randles-Sevcik equation to calculate turnover frequency. Int. J. Hydrogen Energy.

[66] Minakshi M., Mitchell D., Jones R., Alenazey F., Watcharatharapong T., Chakraborty S., Ahuja R. (2016). Synthesis, structural and electrochemical properties of sodium nickel phosphate for energy storage devices. Nanoscale.

[67] Liu J.G., Zhao T.S., Chen R., Wong C.W. (2005). The effect of methanol concentration on the performance of a passive DMFC. Electrochem. Commun..

[68] Barakat N.A.M. (2018). CoNi/CNTs composite as effective and stable electrode for oxygen evaluation reaction in alkaline media. Int. J. Hydrogen Energy.

[69] Abd El-Lateef H.M., Almulhim N.F., Alaulamie A.A., Saleh M.M., Mohamed I.M.A. (2020). Design of ultrafine nickel oxide nanostructured material for enhanced electrocatalytic oxidation of urea: Physicochemical and electrochemical analyses. Colloids Surf. A Physicochem. Eng. Asp..

[70] Barakat N.A., El-Newehy M., Al-Deyab S.S., Kim H.Y. (2014). Cobalt/copper-decorated carbon nanofibers as novel non-precious electrocatalyst for methanol electrooxidation. Nanoscale Res. Lett..

[71] Ghouri Z.K., Barakat N.A.M., Obaid M., Lee J.H., Kim H.Y. (2015). Co/CeO2-decorated carbon nanofibers as effective non-precious electro-catalyst for fuel cells application in alkaline medium. Ceram. Int..

[72] Ghouri Z.K., Barakat N.A.M., Kim H.Y., Park M., Khalil K.A., El-Newehy M.H., Al-Deyab S.S. (2016). Nano-engineered ZnO/CeO2 dots@CNFs for fuel cell application. Arab. J. Chem..

[73] Ghouri Z.K., Barakat N.A.M., Park M., Kim B.-S., Kim H.Y. (2015). Synthesis and characterization of Co/SrCO3 nanorods-decorated carbon nanofibers as novel electrocatalyst for methanol oxidation in alkaline medium. Ceram. Int..

[74] Sarkar C., Nath J., Bhuyan S., Dolui S.K. (2019). Multifunctional ternary nanocomposites of Ni/Polypyrrole/Reduced graphene oxide as supercapacitor and electrocatalyst in methanol oxidation. ChemistrySelect.

[75] Mohamed I., Khalil K.A., Mousa H.M., Barakat N.A. (2017). Ni/Pd-Decorated carbon NFs as an efficient electrocatalyst for methanol oxidation in alkaline medium. J. Electron. Mater..

[76] Perales-Rondón J.V., Solla-Gullón J., Herrero E., Sánchez-Sánchez C.M. (2017). Enhanced catalytic activity and stability for the electrooxidation of formic acid on lead modified shape controlled platinum nanoparticles. Appl. Catal. B Environ..

[77] Yasin A.S., Mohamed I.M.A., Park C.H., Kim C.S. (2018). Design of novel electrode for capacitive deionization using electrospun composite titania/zirconia nanofibers doped-activated carbon. Mater. Lett..

[78] Zhan W., Ma L., Gan M., Xie F. (2022). Ultra-fine bimetallic FeCoP supported by N-doped MWCNTs Pt-based catalyst for efficient electrooxidation of methanol. Appl. Surf. Sci..

[79] Minakshi M., Mitchell D.R., Munnangi A.R., Barlow A.J., Fichtner M. (2018). New insights into the electrochemistry of magnesium molybdate hierarchical architectures for high performance sodium devices. Nanoscale.

[80] Guo F., Ye K., Du M., Huang X., Cheng K., Wang G., Cao D. (2016). Electrochemical impedance analysis of urea electro-oxidation mechanism on nickel catalyst in alkaline medium. Electrochim. Acta.

