# Supplementary material for: Electrocatalysis of Methanol Oxidation in Alkaline Electrolytes over Novel Amorphous Fe/Ni Biphosphate Material Prepared by Different Techniques
Source: Nanomaterials (Basel). 2022 Sep 30;12(19):3429. doi: 10.3390/nano12193429 (PMC9565568; doi:10.3390/nano12193429)
Supplement: Supplementary file 1 [file nanomaterials-12-03429-s001.zip › nanomaterials-1935858-supplementary.pdf]

nanomaterials

MDPI

Supplementary Materials

Electrocatalysis of Methanol Oxidation in Alkaline Electrolytes over Novel Amorphous Fe/Ni Biphosphate Material Prepared by Different Techniques

Mai M. Khalaf [1,2], Hany M. Abd El-Lateef [1,2,*], Van-Duong Dao [3,*] and Ibrahim M. A. Mohamed [2]

[1] Department of Chemistry, College of Science, King Faisal University, Al-Ahsa 31982, Saudi Arabia
[2] Department of Chemistry, Faculty of Science, Sohag University, Sohag 82524, Egypt
[3] Faculty of Biotechnology, Chemistry and Environmental Engineering, Phenikaa University, Hanoi 10000, Vietnam
* Correspondence: hmahmed@kfu.edu.sa or hany_shubra@science.sohag.edu.eg (H.M.A.E.-L.); duong.daovan@phenikaa-uni.edu.vn (V.-D.D.)

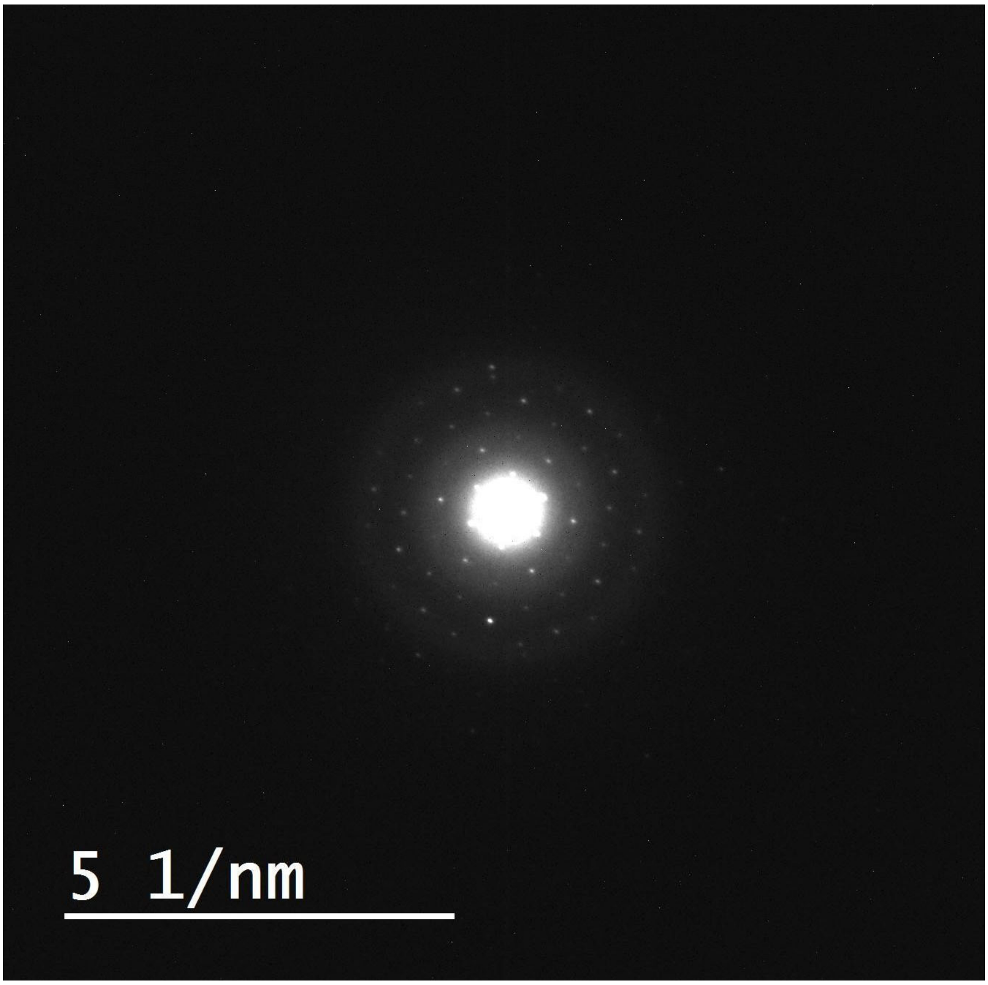

Figure S1. SAED of the synthesized FeNiP-R material.

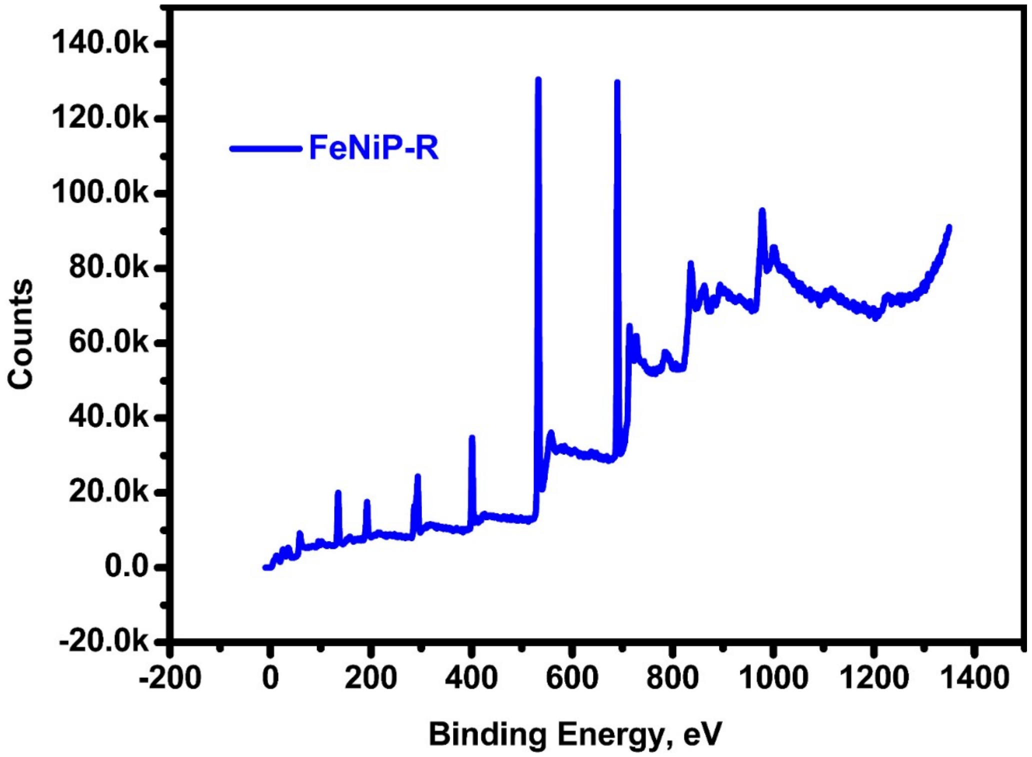

Figure S2. Total survey XPS of the prepared FeNiP-R material in the range of 0–1400 eV.

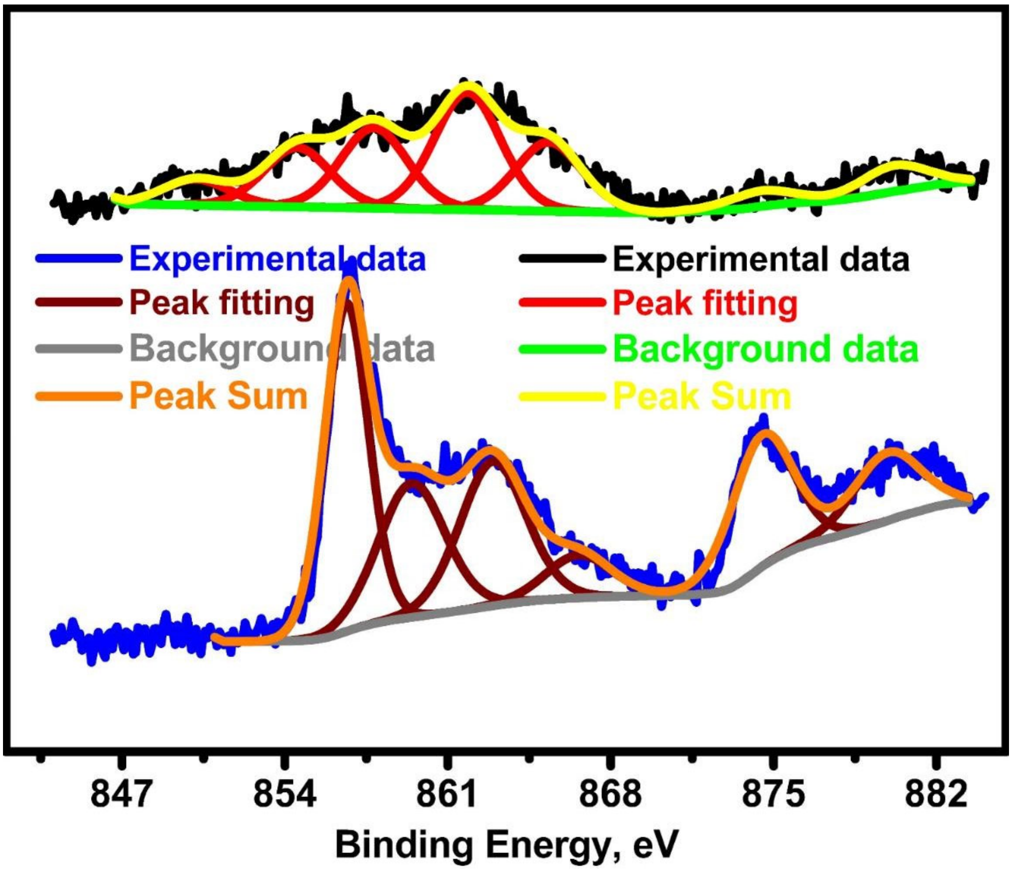

Figure S3. XPS fine spectra of the fabricated FeNiP-R and FeNiP-S materials in the nickel region.

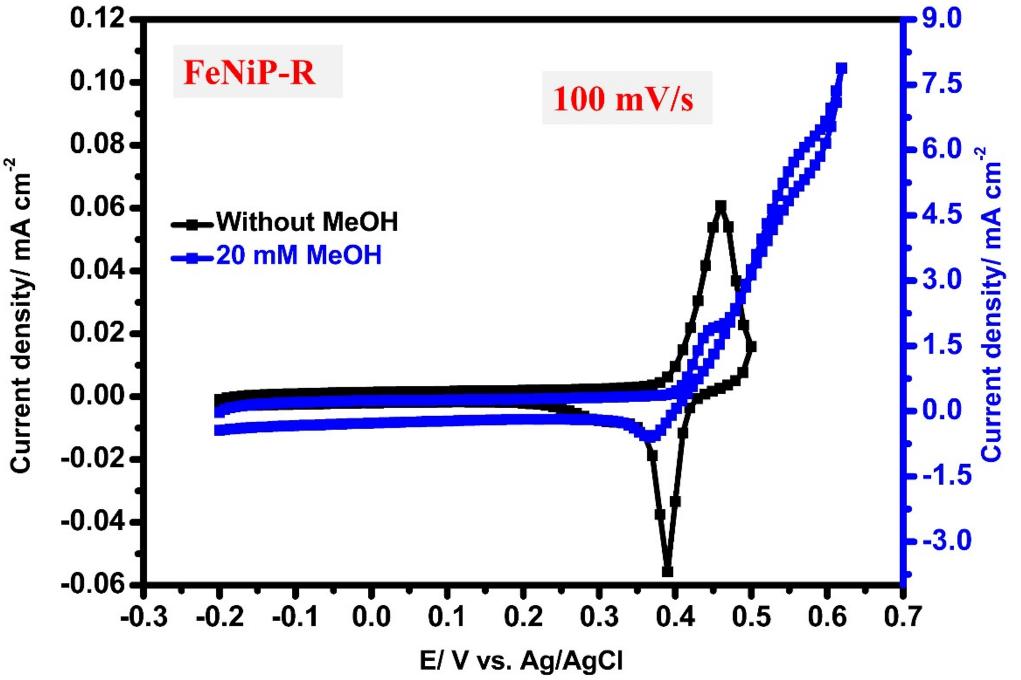

Figure S4. Cyclic voltammograms of the FeNiP-R working electrode in KOH medium with and without 20 mM methanol at 100 mV/s.

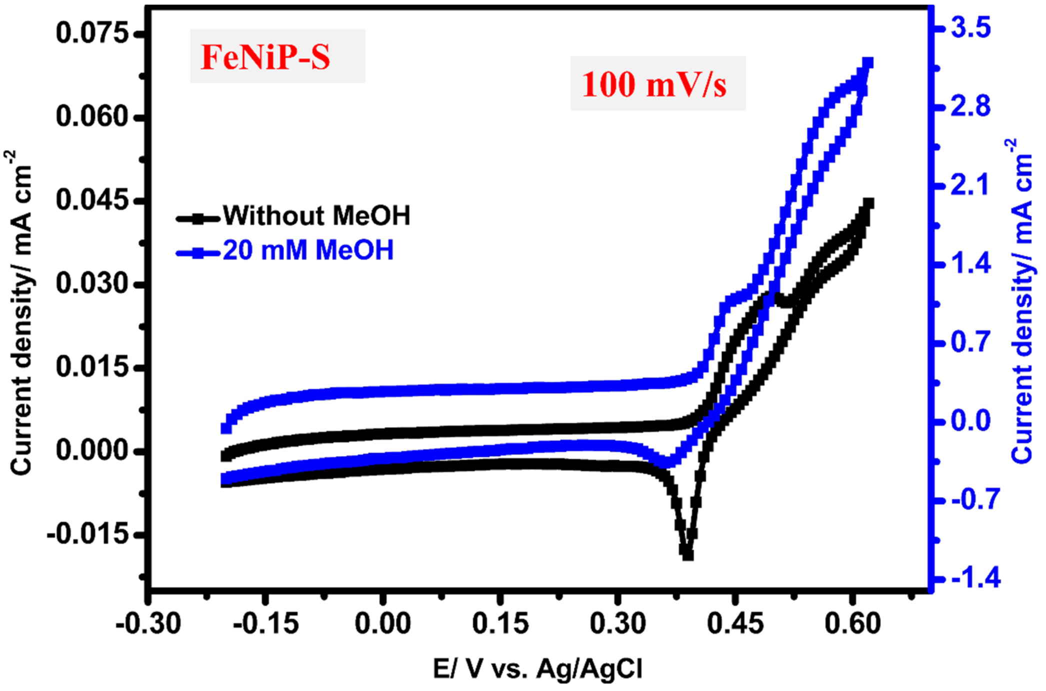

Figure S5. Cyclic voltammograms of the FeNiP-S working electrode in KOH medium with and without 20 mM methanol at 100 mV/s. 37

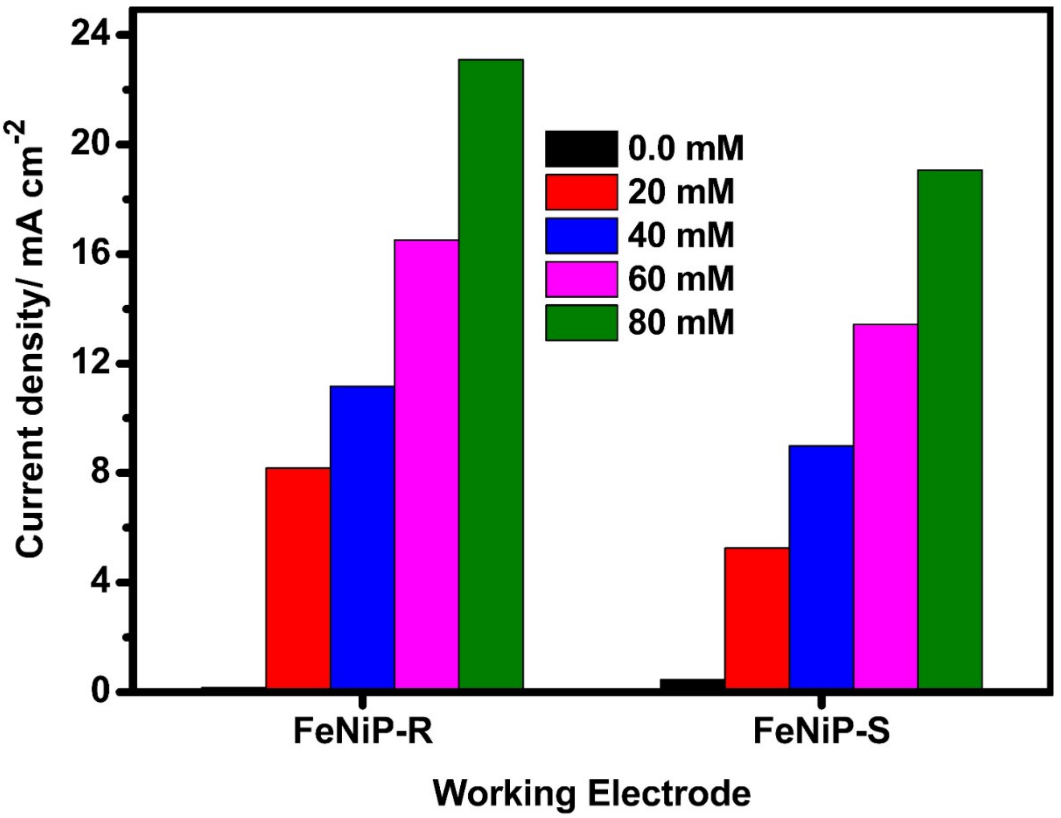

Figure S6. The obtained current values of FeNiP-R and FeNiP-S working electrodes after 1016 s.
